# Non-ischemic Cardiomyopathy: A Rare Adverse Effect of Clozapine

**DOI:** 10.7759/cureus.7901

**Published:** 2020-04-30

**Authors:** Abhinav Garg, Anandbir S Bath, Jagadeesh K Kalavakunta

**Affiliations:** 1 Internal Medicine, Western Michigan University Homer Stryker M.D. School of Medicine, Kalamazoo, USA; 2 Cardiology, Ascension Borgess Hospital, Kalamazoo, USA

**Keywords:** cardiomyopathy, heart failure, clozapine

## Abstract

Clozapine is a dibenzodiazepine antipsychotic used for treatment-resistant schizophrenia. Its association with several side effects such as agranulocytosis, seizure, and insulin resistance is well known. Cardiac side effects such as myocarditis and cardiomyopathy are less common and have been seldom reported. Here we report an unusual case of clozapine-induced nonischemic dilated cardiomyopathy. A 50-year-old female with treatment-resistant schizophrenia on clozapine presented with gradually worsening shortness of breath, productive cough, and pleuritic chest pain. She was found to have non-ischemic dilated cardiomyopathy due to clozapine use as no other causative factor was found. Clozapine was gradually tapered and then discontinued. Repeat echocardiogram in three months revealed improvement in ejection fraction. This case is unique as it outlines clozapine as a rare cause of nonischemic cardiomyopathy, as discontinuation of the drug showed improvement in symptoms and heart function.

## Introduction

Clozapine is a dibenzodiazepine antipsychotic used for treatment-resistant schizophrenia. It is known to reduce positive symptoms such as hallucinations and delusions, improve apathy and social withdrawal, decrease hospital admission, and also reduce mortality due to psychiatry related deaths [1]. Its association with several side effects such as agranulocytosis, seizure, and insulin resistance is well known and monitored. Cardiac side effects such as myocarditis and cardiomyopathy are less common and have been seldom reported. Here we report an unusual case of clozapine-induced nonischemic dilated cardiomyopathy.

## Case presentation

A 50-year-old female with a past medical history of hypertension, hyperlipidemia, tobacco abuse, and multiple psychiatric issues presented to the emergency department with complaints of gradually worsening shortness of breath, productive cough, and left anterior chest pain that gets worse with coughing and certain positions. The patient also complained of orthopnea and the inability to lay flat. The psychiatric history was significant for treatment-resistant schizophrenia currently being treated with clozapine. The patient was tachypneic and hypoxic on presentation. Physical examination revealed normal heart sounds with no murmurs or gallops. Lab workup showed brain natriuretic peptide (BNP) 3200 ng/L, hyponatremia 131 mEq/L, and negative troponins. Electrocardiogram showed sinus bradycardia with first-degree atrioventricular block. Transthoracic echocardiogram showed severe global hypokinesis of the left ventricle with ejection fraction 20% to 25% and moderately dilated left ventricle (Video 1).

**Video 1:** Echocardiography showing reduced ejection fraction

The patient underwent cardiac catheterization which revealed nonobstructive coronary arteries with severe nonischemic cardiomyopathy. No other causative factor could be identified causing this cardiomyopathy. Her urine drug screen was negative for alcohol and cocaine which are common causes for dilated cardiomyopathy. Her antinuclear antibody (ANA) was negative as well. Her medications were reviewed, and Psychiatry was consulted to evaluate for clozapine as a cause of nonischemic cardiomyopathy. Clozapine was gradually tapered and then discontinued after discussion with psychiatry. The patient was placed on guideline-directed medical therapy for heart failure with reduced ejection fraction. The patient reported improvement in her shortness of breath, chest pain, and orthopnea at a three-month cardiology follow-up. Repeat echocardiogram revealed an improved ejection fraction of 45% to 50% (Video 2).

**Video 2:** Echocardiography showing improved ejection fraction

This case is unique as it outlines clozapine as a rare cause of nonischemic cardiomyopathy, as discontinuation of the drug showed improvement in symptoms and heart function.

## Discussion

Clozapine is a dibenzodiazepine antipsychotic that is Food and Drug Administration (FDA) approved for treatment-resistant schizophrenia for patients who have failed conventional therapy with at least two different antipsychotics. Clozapine is associated with several well-known side effects such as agranulocytosis, seizure, insulin resistance and some lesser-known cardiac side effects such as myocarditis and cardiomyopathy. It can also cause pericardial effusion as part of cardiotoxicity [2].

Per national database reports, the incidence of clozapine-induced cardiomyopathy is about 8.9 per 100,000 person-years [3]. The pathophysiology of clozapine-induced cardiomyopathy is not well defined but various theories exist. Cardiomyopathy that develops within one month of initiation of the drug along with peripheral eosinophilia suggests an underlying type one immunoglobulin E (IgE) mediated hypersensitivity reaction. Peripheral eosinophilia was not present in our case. There could also be a direct toxic effect on the heart similar to what has been observed in anthracycline-induced cardiotoxicity. Some autopsy reports have shown evidence of eosinophilic infiltrates on endomyocardial biopsy [4]. Eosinophilia is not known to be a reliable predictor of underlying cardiomyopathy. Cardiomyopathy can occur as sequelae of underlying myocarditis which would explain the delayed manifestation of cardiomyopathy several months to years after drug initiation. Most of the cases occurred at standard doses or even at low doses suggesting a non-dose dependent effect of clozapine cardiotoxicity [5].

Patients can present with nonspecific signs such as fever, fatigue, palpitations, acute chest pain, or overt signs of heart failure such as orthopnea, leg swelling, shortness of breath [6]. Clozapine-induced cardiomyopathy can occur as soon as three weeks after initiation of therapy till up to four years of continuous use of the drug but most cases occur within the first month of drug initiation. Our patient was on clozapine for about eight months before the presentation. The myocardial disease can present as tachycardia and patients with persistent or new tachycardia should be evaluated for cardiomyopathy. Reduced ejection fraction on echocardiogram can confirm the diagnosis with dilated cardiomyopathy being the most common finding. The electrocardiogram can show signs of QT prolongation [7]. Troponin elevation can be seen in patients with underlying myocarditis.

Agranulocytosis is a well-known side effect of clozapine and thus there are well-documented guidelines to monitor it. Cardiomyopathy and myocarditis are such rare adverse effects that routine monitoring is not done. Patients with pre-existing cardiac history or risk factors for cardiomyopathy (advanced age, poorly controlled hypertension or diabetes, hyperlipidemia, and active smoker) should be monitored more closely, especially for the first few weeks. A baseline electrocardiogram should be done to monitor for QT prolongation. Consider C reactive protein and troponin monitoring for patients with cardiac disease [8].

Ronaldson et al. (2011) came up with a monitoring protocol for clozapine-induced cardiotoxicity which included baseline troponin, C reactive protein, and transthoracic echocardiography. This is accompanied by the assessment of biomarkers weekly, vital signs every other day, and symptoms of illness daily. Clozapine can be continued if troponin is less than twice the upper limit of normal or C reactive protein is less than 100 mg/L [9]. However, baseline transthoracic echocardiography could be a major limitation to initiating clozapine therapy especially in areas with limited healthcare and might not be cost-effective.

Clozapine should be discontinued in patients with suspected or proven cardiomyopathy. A cardiology consultation should be considered with a follow-up transthoracic echocardiography to ensure the remission of cardiomyopathy and normalization of ejection fraction. In our patient, ejection fraction improved significantly on repeat echocardiography at three months. In patients with reduced ejection fraction, disease-modifying heart failure medications such as beta-blockers, angiotensin-converting enzyme inhibitors or mineralocorticoid receptor antagonists should be initiated until remission is achieved [10].

## Conclusions

Symptoms of heart failure with no identifiable cause in a patient on clozapine should prompt evaluation for cardiac side effects from the drug. Unlike for agranulocytosis, a regular monitoring program does not exist for cardiac side effects. A cardiology consultation should be considered if suspicion for clozapine-induced cardiomyopathy is high. Discontinuation of clozapine after discussion with psychiatry and initiation of heart failure therapy is the mainstay of treatment. Hence, this case is unique as it outlines clozapine as a rare cause of nonischemic cardiomyopathy, as discontinuation of the clozapine in our patient showed improvement in symptoms and heart function.
